# Extracellular Vesicles (Secretomes) from Human Trophoblasts Promote the Regeneration of Skin Fibroblasts

**DOI:** 10.3390/ijms22136959

**Published:** 2021-06-28

**Authors:** Yoon Young Go, Chan Mi Lee, Won Min Ju, Sung-Won Chae, Jae-Jun Song

**Affiliations:** 1 Department of Otorhinolaryngology-Head and Neck Surgery, Korea University Guro Hospital, Seoul 08308, Korea; gokogoko@korea.ac.kr (Y.Y.G.); cksal7873@korea.ac.kr (C.M.L.); juwonmin22@naver.com (W.M.J.); chaeorl@korea.ac.kr (S.-W.C.); 2 Institute for Health Care Convergence Center, Korea University Guro Hospital, Seoul 08308, Korea

**Keywords:** human trophoblasts, extracellular vesicles, exosomes, skin regeneration, aging, chemokines

## Abstract

To date, placental trophoblasts have been of interest in the fields of obstetrics and gynecology, mainly due to their involvement in the formation of a connection between the mother and fetus that aids in placental development and fetal survival. However, the regenerative capacities of trophoblasts for application in regenerative medicine and tissue engineering are poorly understood. Here, we aim to determine the skin regeneration and anti-aging capacities of trophoblast-derived conditioned medium (TB-CM) and exosomes (TB-Exos) using human normal dermal fibroblasts (HNDFs). TB-CM and TB-Exos treatments significantly elevated the migration and proliferation potencies of HNDF cells in a dose- and time-dependent manner. When RNA sequencing (RNA-seq) was used to investigate the mechanism underlying TB-CM-induced cell migration on scratch-wounded HNDFs, the increased expression of genes associated with C-X-C motif ligand (CXCL) chemokines, toll-like receptors, and nuclear factor-kappa B (NF-κB) signaling was observed. Furthermore, treatment of intrinsically/extrinsically senescent HNDFs with TB-CM resulted in an enhanced rejuvenation of HNDFs via both protection and restoration processes. Gene expression of extracellular matrix components in the skin dermis significantly increased in TB-CM- and TB-Exos-treated HNDFs. These components are involved in the TB-CM and Exo-mediated regeneration and anti-aging of HNDFs. Thus, this study demonstrated the regenerative and anti-aging efficacies of trophoblast-derived secretomes, suggesting their potential for use in interventions for skin protection and treatment.

## 1. Introduction

The placenta is a unique maternal organ in mammals that exists temporarily for the development of the fetus. After delivery, the placenta is usually discarded, but owing to its multifunctional properties, such as anti-inflammatory, anti-oxidant, anti-bacterial/viral, radioprotective, and immunological modulation, it is well-regarded as a natural biomaterial [1,2,3,4]. Many researchers and clinicians are interested in placenta-derived materials, including placenta extracts, cord blood serum and cells, the amniotic/chorionic membrane, and placental mesenchymal stem cells (MSCs), to study their therapeutic potential and clinical applications in the field of tissue engineering [5]. Of particular interest is the regenerative efficacy of trophoblast cells found in the chorionic membrane. The trophoblast layer occupies most of the chorionic membrane and plays an important role in maternal–fetal communication, which might rely on trophoblast-derived materials such as extracellular vesicles (EVs) [6,7]. However, the therapeutic efficacy of trophoblasts has not been well studied.

Currently, MSCs are still considered the most common cell source in various regenerative medicine strategies for damaged tissue repair and engineering [8,9]. Although MSCs can be obtained easily from adipose tissue, bone marrow, dental pulp, and umbilical cord, they constitute a small population in the whole tissue; the cells thus obtained suffer from a low yield of in vitro expansion and loss of biological activity during long-term culture [10]. These issues consequently impede the development of MSC therapies for regenerative medicine. Therefore, finding alternative cell sources is a pressing need in the field of regenerative medicine, for the development of clinical applications. From this perspective, trophoblasts can serve as a desirable cell candidate with bioactive potential. It has been shown that trophoblasts secrete numerous growth factors, hormones, and biological signal substances containing EVs, such as microvesicles and exosomes, to transmit maternal signals to the fetus [11]. One of the recent approaches in regenerative medicine is cell-free therapy involving cell-derived conditioned medium (CM) and exosomes, for injection into live cells [12]. Despite the potential benefits of MSCs, their use for direct cell therapy may cause undesirable side effects, such as inflammation and tumorigenesis in recipients [10]. Cell-free exosome therapy offers safety strategies, as well as well-established quality control and guidelines for clinical settings [13]. Stem cell-derived CMs and exosomes have been studied for their regenerative effects. Exosomes from MSCs have been studied for their bone and cartilage regenerative effects [14,15]. In addition, Oh et al. studied the skin regeneration effects of human-induced pluripotent stem cell-derived exosomes [16].

We also hypothesized that trophoblasts containing EVs have regenerative effects, and investigated the regenerative effect of trophoblast-derived conditioned medium (TB-CM) and exosomes (TB-Exos) on human skin fibroblasts. The excellent proliferative and anti-aging effects of TB-CM and TB-Exos on human normal dermal fibroblasts (HNDFs) were explored while using MSC-CM and MSC-Exos as controls. The mechanisms underlying TB-CM-induced cell migration during wound healing in HNDFs were also determined using RNA sequencing (RNA-seq) analysis. Additionally, we also showed that TB-derived materials provided skin protection against UV photoaging by recovering UV-induced skin damage and protecting against it, thus serving as effective anti-photoaging agents.

Thus, the present study reveals the possible value of trophoblasts in skin regeneration and demonstrates the therapeutic potential of trophoblast-derived secretomes, such as EVs, for skin rejuvenation, to be considered for an application in clinical medicine and cosmetic manufacturing.

## 2. Materials and Methods

### 2.1. Cell lines and Culture Procedure

The human trophoblast cell line (HTR-8/SVneo) was obtained from American type culture collection (ATCC) (Manassas, VA, USA; CRL-3271) and cultured in RPMI-1640 (Gibco, Grand Island, NY, USA) supplemented with 5% fetal bovine serum (FBS) (Gibco) and penicillin/streptomycin (1000 U/mL, Gibco, New York City, NY, USA). HNDF cells were kindly provided by Dr. Shin Ohk (Department of Infectious Immunology, Korea University Guro Hospital, Seoul, Korea) and maintained in DMEM (Gibco) supplemented with 10% FBS (Gibco) and penicillin/streptomycin (1000 U/mL, Gibco). Human bone marrow MSCs were purchased from Cefobio (Gyeonggi-do, Korea; CEFO-BMMSC) and cultured in MEM (Gibco) supplemented with 5% FBS (Gibco) and penicillin/streptomycin (1000 U/mL, Gibco). All cells were maintained in a humidified atmosphere at 37 °C, with 5% CO_2_.

### 2.2. Preparation of Conditioned Medium and Exosomes

When the confluency of trophoblast cells reached 50%, the growth medium was replaced with 5% (*v*/*v*) exosome-depleted FBS (Gibco) and the cells were incubated in it for 48–72 h until cell confluency reached 100%, post which the CM was collected. The CM was then filtered with a 0.2 μm syringe filter (Millipore, Billerica, MA, USA) before use. Exosomes were isolated from the collected CM using an exosome isolation kit (ExoQuick-TC™, Systemic Biosciences, Palo Alto, CA, USA), according to the manufacturer’s protocol. Briefly, CM was centrifuged at 1000× *g* for 5 min to remove the cells and filtered using a 0.22 μm syringe filter. The ExoQuick-TC™ solution was added to the supernatant in a ratio of 1:5, mixed carefully, and then left at 4 °C for 16 h. The exosomal pellets were precipitated by means of centrifugation at 3000× *g* for 30 min, resuspended in phosphate buffered saline (PBS), and used immediately or stored at −80 °C until use.

### 2.3. Nanoparticle Tracking Analysis

The size and concentration of the TB-Exos were determined using a NanoSight™ LM10-HS10 system (NanoSight, Amesbury, UK). Exosome samples were diluted with PBS at a ratio of 1:10 and three recordings were performed in 30 s. A combination of a monochromatic laser beam (405 nm) and NanoSight™ tracking software version 3.0 were used to analyze the average exosome size and concentration.

### 2.4. Western Blot Analysis

TB-Exos were lysed and the same amounts of proteins were subjected to immunoblotting using antibodies against CD9, CD63, CD81, ALIX (used at a ratio of 1:1000; Systemic Biosciences), and β-actin (used at a ratio of 1:2000; Santa Cruz Biotechnology, Dallas, Texas, USA). Immunoreactive protein bands were captured using a Fusion Solo Imaging System (Vilber Lourmat, Marne-la-Vallée, France).

### 2.5. CCK8 Assay

HNDFs were seeded at a density of 1 × 10^4^ cells/well into 96-well plates and then treated with TB-CM or TB-Exo in serum-free medium for 24 or 72 h, under the indicated conditions. Cell proliferation was measured using a CCK8 kit (Dojindo Laboratories, Kumamoto, Japan), according to the manufacturer’s protocol. Absorbance of the live cells was measured at 450 nm using a microplate spectrophotometer.

### 2.6. Wound Scratch Assay

HNDFs were seeded into 6-well plates at a density of 5 × 10^6^ cells per well and incubated at 37 °C in 5% CO_2_. After incubation for 24 h, the cells were scratched at regular intervals using a sterilized 200-μL pipette tip. The cells were washed with PBS, following which TB-CM or TB-Exos were added to them. Images of the scratched areas from three independent experiments were observed at 0, 24, and 48 h using a cellSens imaging camera (ver.1.18) and software. The area of cell migration was quantified using ImageJ software, calculating the ratio of the scratch area at the given point in time and the original scratched area, 0 h. At least three-microscope fields were determined for each condition.

### 2.7. SA-β-Gal Staining

Senescent HNDFs from passages 19–22 were seeded into 6-well plates at a density of 1 × 10^6^ cells/well and incubated for 24 h. Following that, 20% TB-CM or MSC-CM was added to the cells and the cells were incubated with it for 72 h. The cells were then stained with SA-β-Gal using a Senescence Cells Histochemical Staining Kit (Sigma-Aldrich, St. Louis, MO, USA), and three images were obtained from each group. Strong, weak, and unstained cells upon SA-β-Gal staining were classified into groups and the cells in each group were counted.

### 2.8. RNA Extraction and Quantitative Real-Time RT-PCR

RNA was extracted using TRIzol™ reagent (Invitrogen, Carlsbad, CA, USA) to analyze the relative gene expression. PrimeSript™ 1st strand cDNA Synthesis Kit (Takara Bio, Tokyo, Japan) was used for the reverse transcription of 1 μg of total RNA to cDNA, according to the manufacturer’s instructions. PCR was then carried out using the obtained cDNA as a template with the Power^®^ SYBR Green PCR Master Mix (Life Technologies Co. Ltd., Woolston Warrington, UK). The standard PCR cycle conditions were as follows: 95°C for 1 min, 40 cycles of denaturation at 95 °C for 15 s, and annealing-extension at 60 °C for 30 s. To analyze the relative expression level of the mRNA, the 2^(^^−∆∆C*t*)^ method was used, with normalization to *GAPDH*. The specific primer sequences used in this study are listed in Table 1.

### 2.9. Ultraviolet B Irradiation on HNDFs

Senescent HNDFs were seeded at a density of 2 × 10^5^ cells/well into 6-well plates and incubated in growth medium, until the confluence reached 70%. The cells were then washed with PBS and exposed to ultraviolet B radiation for 40 s, at a dose of 0.36 J/cm^2^, using a UV transilluminator (Bi-O-Vision™ UV transilluminator, SpectroLine, NY, USA) with a spectral peak at 312 nm. UV irradiation was performed before and after treatment with TB-CM. After 72 h, SA-β-Gal staining was performed, and stained/unstained cells were examined.

### 2.10. RNA-seq

The concentration and purity of the isolated RNA were determined using Quant-iT™ RiboGreen™ Assay Kit (Invitrogen, #R11490). High-quality mRNA for Illumina libraries were prepared with RNA integrity number (RIN) greater than 7.0, according to the TruSeq^®^ Stranded mRNA Sample Preparation Guide (Illumina platform, part number 15031047 Rev. E). The transcriptome library was constructed using the TruSeq^®^ Stranded mRNA LT Sample Prep Kit (Illumina, San Diego, CA, USA), according to the manufacturer’s protocol. RNA fragments (1000 ng) from each group were purified using poly T-attached magnetic beads and then reverse-transcribed to cDNA using SuperScript™ II Reverse Transcriptase (Invitrogen, #18064014). Adapters were ligated to the library and amplified using PCR, to generate the final cDNA library. The concentrated library was quantity- and quality-controlled using KAPA Library Quantification Kits for Illumina Sequencing platforms, according to the qPCR Quantification Protocol Guide (Kapa Biosystems, MA, USA, #KK4854) and the TapeStation D1000 ScreenTape (Agilent Technologies, Santa Clara, CA, #5067-5582), following the manufacturer’s instructions. Multiple libraries were combined in equal molar ratios and then sequenced on an Illumina NovaSeq platform (Illumina).

Adapter and low-quality sequences were removed before analysis of the transcriptome target regions, following which the quality-controlled sequences were read with the *Homo sapiens* (GRCh38) sequences using HISAT v2.1.0 [17]. The reference genome sequences and annotations for *Homo sapiens* (GRCh38) were obtained from the UCSC genome browser (http://genome.uscs.edu, accessed on 31 March 2021). After alignment with HISAT, the relative abundance of the transcript-level expression was analyzed in terms of read count values using StringTie v2.1.3b [18,19]. To determine the DEGs, statistical analyses such as the Benjamini-Hochberg algorithm were used to obtain adjusted *p*-values < 0.05. Gene ontology, functional annotation, and enrichment pathways for significant genes were analyzed using gProfiler (https://biit.cs.ut.ee/gprofiler/gost, accessed on 5 April 2021) and KEGG.

### 2.11. Statistical Analysis

Statistical analyses were performed using Student’s two-tailed *t*-test and 2-way ANOVA with Prism 7 software (GraphPad, San Diego, CA, USA). The *p*-values are shown in the figures. The differences were considered statistically significant at * *p* < 0.05, ** *p* < 0.01, and *** *p* < 0.001. All representative data were obtained from triplicate experiments and are presented as mean ± standard deviation.

## 3. Results

### 3.1. Characterization of Extracellular Vesicles Derived from Human Trophoblasts

To investigate the regenerative effects of human trophoblast-derived biological factors on human dermal fibroblasts, we first prepared conditioned medium and exosomes from trophoblasts. Conditioned medium secreted by human trophoblast cells was obtained at 50–100% confluency of cells. Nanoparticle tracking analysis and Western blotting were carried out to characterize the exosomes secreted from human trophoblasts using size and exosome-specific marker proteins. The average size of exosomes isolated from trophoblasts was 132.2 ± 3.9 nm, which is within the known range of exosome size (30–150 nm) for small membrane vesicles [20]. EVs contained in TB-CM showed a broader size distribution than TB-Exos, with an average EV size of 195 ± 4.3 nm (Supplementary Figure S1A). Exosome-specific markers, such as CD9, CD63, CD81, and ALIX, were detected in exosomes from trophoblasts using Western blotting (Supplementary Figure S1B).

### 3.2. TB-CM and TB-Exos Enhanced the Proliferation of HNDFs

**Figure 1:**
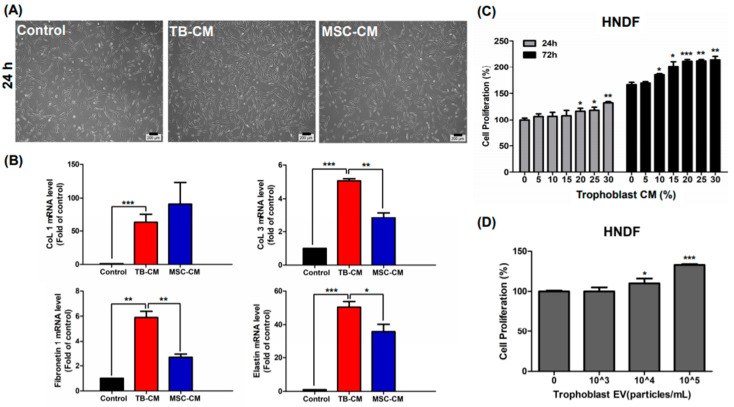
TB-CM and TB-Exos induced proliferation of HNDFs. (**A**) HNDFs were treated with 20% TB-CM and MSC-CM for 24 h, followed by the estimation of the number of cells under a light microscope. Scale bar: 200 μm. (**B**) After treatment of HNDFs with 20% TB-CM and MSC-CM for 24 h, the cells were harvested and the relative mRNA levels of collagen type I and III, fibronectin 1, and elastin in the cells were quantified using quantitative RT-PCR analysis. (**C**) HNDF cells were treated with 0%, 5%, 10%, 15%, 20%, 25%, and 30% TB-CM, respectively. The cell viability was measured using CCK8 assay at 24 and 72 h. (**D**) HNDF cells seeded into a 96-well plate were treated with TB-Exos (1 × 10^3^, 1 × 10^4^, and 1 × 10^5^ particles/mL), following which the cells were cultured in growth medium for 24 h. Cell proliferation was determined using CCK8 assay. Data are represented as means and standard deviation (n = 3); * *p* < 0.05, ** *p* < 0.01, and *** *p* < 0.001, as compared to the corresponding control.

We first compared the effect of TB-CM on proliferation with that of MSC-derived conditioned medium (MSC-CM), because MSC-CM is a well-known trophic activator in the field of regenerative medicine [21,22]. Our results indicated that exogenous treatment with TB-CM for 24 h highly induced the proliferation of HNDFs, as compared to treatment with MSC-CM (Figure 1A). Quantitative real-time polymerase chain reaction analysis showed that treatment of HNDFs with TB-CM led to an increase in the relative expression of genes such as *collagen type 1 and 3*, *elastin*, and *fibronectin* (Figure 1B), which are related to the constitution of the dermal matrix as a structural component of the skin dermis [23]. We analyzed the effect of cell proliferation on HNDFs using Cell Counting Kit 8 (CCK8) assay post-treatment with various concentrations of TB-CM (0–30%) for 24 and 72 h. A greater increase in proliferation was detected in HNDF cells cultured with TB-CM (< 20%) than in the untreated cells (Figure 1C). In addition, proliferation of HNDFs was significantly promoted by TB-CM in a time-dependent manner upon treatment for 24 h to 72 h, indicating excellent efficacy. Similar to the CCK8 assay results, cell counting assay results also showed the stimulatory effect on HNDF cell proliferation at 24, 48, and 72 h post-treatment with different concentrations of TB-CM, suggesting that TB-CM has cell proliferation potential in HNDFs (Supplementary Figure S2A). No cytotoxic effect was observed, even in the group treated with high amounts of TB-CM (60%). TB-Exo also displayed a positive effect on the proliferation of HNDFs; treatment with 1 × 10^4^ and 1 × 10^5^ particles/mL of TB-Exo caused a dose-dependent increase in the proliferation of HNDFs (10% and 30%, respectively; Figure 1D). These results demonstrated that TB-CM and TB-Exos have non-cytotoxic effects on HNDFs.

### 3.3. TB-CM and TB-Exos Have Migration-Promoting Effects on HNDFs

**Figure 2:**
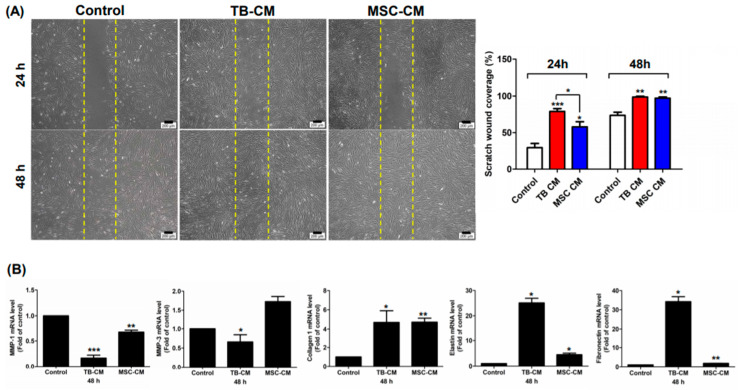
TB-CM induced the migration of HNDFs. (**A**) HNDF cells were scratched and then treated with 20% TB-CM and/or MSC-CM in serum-free media. After incubation for 24 and 48 h, the migrated cells were stained and visualized under a light microscope. Scale bar: 200 μm. The area of migration was quantified for each group using ImageJ and is represented as a graph. (**B**) The expression levels of genes encoding for MMP-1, MMP-3, collagen type I, fibronectin 1, and elastin were determined using quantitative real-time RT-PCR post-treatment of scratch-wounded HNDFs with 20% TB-CM and MSC-CM for 48 h. Data are represented as means and SD; * *p* < 0.05, ** *p* < 0.01 and *** *p* < 0.001, as compared to the corresponding control.

**Figure 3:**
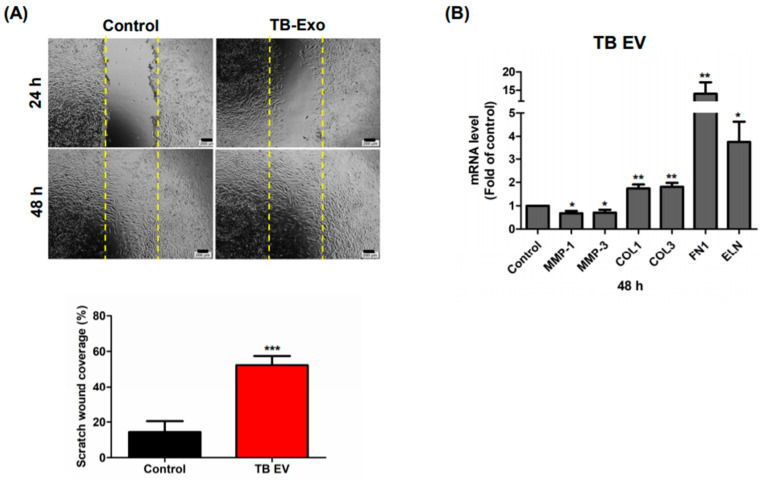
TB-Exos induced migration of HNDFs. (**A**) Scratch-wounded HNDF cells were treated with 1 × 10^5^ particles/mL of TB-Exos for 24 and 48 h, followed by the determination of the area of cell migration using ImageJ. Scale bar: 200 μm. (**B**) Quantitative RT-PCR method was used to analyze the relative expression levels of genes encoding for MMP-1, MMP-3, collagen type I, fibronectin 1, and elastin in TB-Exo-treated scratch-wounded HNDFs. Data are represented as means and SD; * *p* < 0.05, ** *p* < 0.01, and *** *p* < 0.001, as compared to the control.

In the next part of the study, we investigated whether TB-CM and TB-Exos stimulated the migration of HNDFs. In the scratch wound assays, scratched HNDFs were treated with TB-CM and TB-Exos, following which the coverage rate of the scratched area was evaluated. MSC-CM-treated and untreated HNDF cells were used in this assay as positive and negative controls, respectively. The rate of migration of the HNDF cells increased rapidly upon TB-CM treatment at 24 h, as compared to that of the control HNDF cells, and covered 80% of the scratch-wounded area (Figure 2A). MSC-CM also promoted the migration of HNDFs, but closed the scratched area gradually, in a time-dependent manner. In addition, the expression levels of skin regeneration-related genes, including genes encoding for matrix metalloproteinase-1 (*MMP-1*), matrix metalloproteinase-3 (*MMP-3*), collagen type 1, elastin, and fibronectin were evaluated when scratch-wounded HNDFs were cultured with or without TB-CM and MSC-CM (Figure 2B). As compared to the untreated HNDFs, cells treated with TB-CM displayed a significant decrease in the mRNA levels of *MMP-1* and *MMP-3*, which have an inhibitory role in the regenerative capacity of skin as extracellular matrix proteases. Increased collagen, elastin, and fibronectin gene expression were determined in TB-CM-treated HNDFs, indicating that TB-CM is capable of upregulating the gene expression of these important structural proteins in the dermis. Treatment of HNDFs with MSC-CM caused an increase in the expression of collagen-, elastin-, and fibronectin-encoding genes, but the fold change increase in the expression of these genes was higher in the TB-CM-treated HNDFs (collagen, 6-fold; elastin, 25-fold; fibronectin, 35-fold) than in the MSC-CM-treated cells (collagen, 5.5-fold; elastin, 5-fold; fibronectin, 2.5-fold). The migration-stimulatory effect of TB-Exos was also analyzed in this protocol, indicating that the migration rate of HNDF cells increased significantly (by 3-fold) in cells treated with TB-Exo (Figure 3A). Treatment with TB-Exo also led to similar results to those of the treatment with TB-CM; there was a decrease in the mRNA levels of *MMP-1* and *MMP-3*, while the expression levels of collagen, elastin, and fibronectin increased significantly in the TB-Exo-treated HNDFs, as compared to in the untreated cells (Figure 3B). These results demonstrated that TB-CM and TB-Exos significantly induced the migration and reconstitution capacity of HDNFs in the scratch-wounded area, as compared to the control cells.

### 3.4. TB-CM Recovered the Senescence of HNDFs

**Figure 4:**
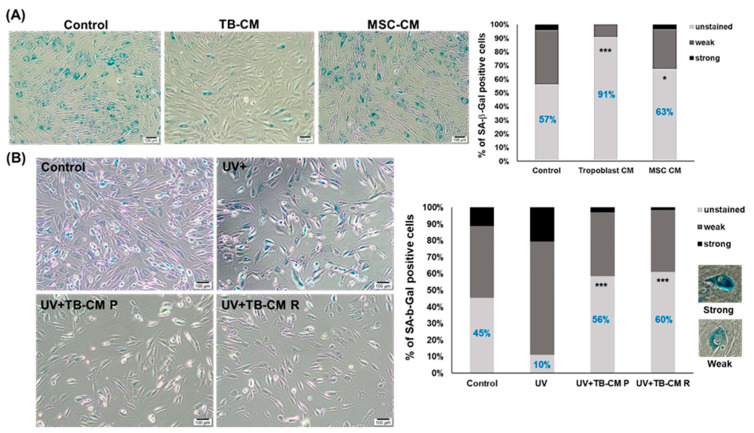
Anti-aging effect of TB-CM on senescence in HNDFs. (**A**) Senescent HNDF cells (at passage 21) were treated with 20% TB-CM and MSC-CM for 72 h and then stained with SA-β-Gal. Positive staining with SA-β-Gal was detected in terms of strong and weak blue color upon observation under a light microscope. According to the degree of SA-β-Gal stain on the cells, the cells were divided into unstained, weak, and strong groups, for the quantification of the stained cells. The percentage of SA-β-Gal-positive cells was determined in three independent images. Scale bar: 100 μm. (**B**) Senescent HNDF cells were incubated with TB-CM (20%) before and after UV irradiation, and the number of cells positively stained with SA-β-Gal was determined in three different images in each group. Scale bar: 100 μm. Data are represented as means and standard deviation (n = 3); * *p* < 0.05 and *** *p* < 0.001, as compared to the corresponding control. Representative images of strongly and weakly stained cells have been included in the figure.

Next, we validated whether TB-CM reversed the senescence of HNDFs. To assess the aging recovery capacity of TB-CM on HNDFs, senescent HNDF cells were cultured with or without TB-CM and MSC-CM for 24 h. The expression of senescence-associated β-galactosidase (SA-β-Gal) was used in this experiment as a typical senescence marker in aging skin [24]. As shown in Figure 4A, positive staining of SA-β-Gal was highly observed in the senescent HNDFs, but upon treatment with TB-CM, there was a remarkable reduction in the strong expression of SA-β-Gal in these cells. The proportion of SA-β-Gal-positive stained area significantly decreased and disappeared in TB-CM-treated groups, as compared to that in the untreated control cells (unstained area in the untreated group, 57%; TB-CM group, 91%). Treatment with MSC-CM also slightly reduced the ratio of strongly and weakly stained cells, but TB-CM more effectively recovered the senescence of dermal fibroblasts. We also determined the anti-aging effect of TB-CM on UV-induced damage in HNDFs. Extrinsic aging caused by UV in senescent HNDFs resulted in a sharp increase in the number of SA-β-Gal-positive cells; upon treatment with TB-CM, there was recovery in the number of unstained cells in this group (Figure 4B). Upon treatment of senescent HNDFs with TB-CM before and after UV exposure, to investigate the protective and recovery effects of TB-CM on skin fibroblasts, TB-CM was found to have an anti-photoaging effect on HNDFs in both the groups. This result indicated that TB-CM inhibited the senescence of HNDF cells. Moreover, remarkable protection and recovery of aged skin cells were observed in the SA-β-Gal staining analysis when senescent HNDFs were treated with TB-CM, which could indicate that trophoblast-derived substances can be used as excellent anti-aging materials for skin rejuvenation.

### 3.5. Transcriptomic Analysis of TB-CM in Scratch-Wounded HNDFs: Chemokine Signaling Pathway

**Figure 5:**
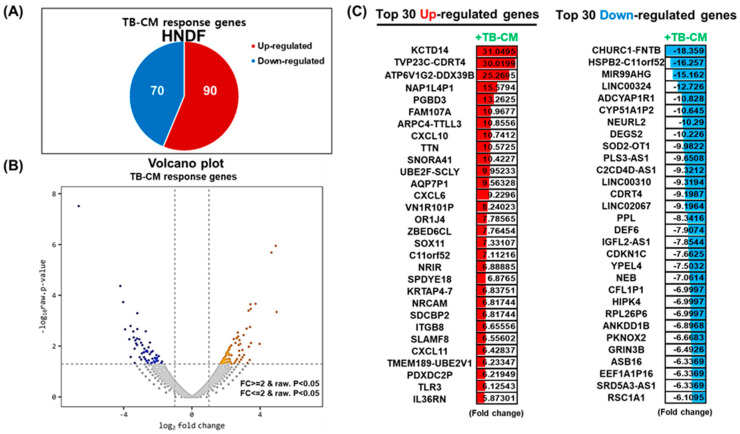
TB-CM induced widespread transcriptome alteration in HNDF cells during migration. (**A**) Scratch-wounded HNDF cells were treated with 20% TB-CM, following which RNA-seq was performed to identify the DEGs upon the treatment of HNDFs with TB-CM. The number of up- and downregulated genes in response to TB-CM treatment of HNDF cells is indicated (*p* < 0.05). (**B**) The distribution of significant DEGs is visualized in the form of a Volcano plot, which analyzed statistical significance versus fold change. (**C**) The top 30 up- and downregulated genes in TB-CM-treated HNDFs are represented by means of color images and value of fold change (fold difference with *p* < 0.05). The color depicts the levels of fold change.

**Figure 6:**
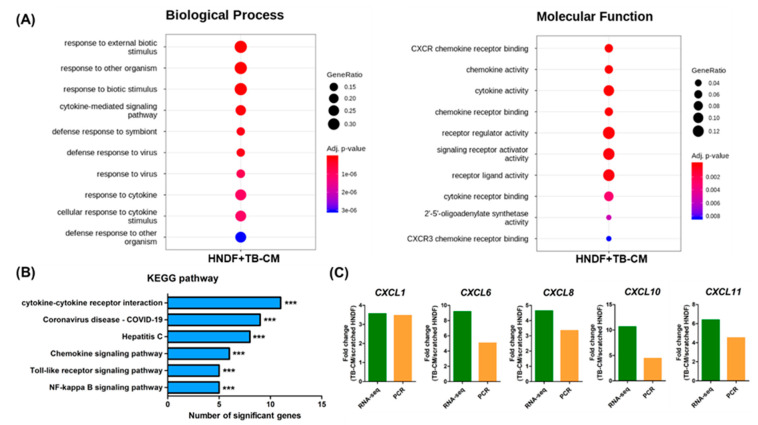
Changes in biological processes in HNDFs upon treatment with TB-CM. (**A**) The top 10 gene ontology terms of associated biological processes and molecular function from DEGs in TB-CM-treated HNDF cells are represented using a dot plot with adjusted *p*-value (*p* < 0.05). GeneRatio is the intersection size/query size, while the dot size represents the intersection size. (**B**) KEGG pathway analysis showed the significantly correlated biological pathways in the results of RNA-seq upon treatment of HNDFs with TB-CM during scratch wound migration. (**C**) The representative upregulated CXCL-related genes, namely *CXCL1*, *CXCL6*, *CXCL8*, *CXCL10*, and *CXCL11*, in TB-CM-treated HNDFs were confirmed using quantitative real-time RT-PCR and compared with the results of RNA-seq.

We next investigated the changes triggered at the whole-transcriptome level upon TB-CM treatment of scratch-wounded HNDFs, to analyze the mechanism of the wound-healing effect of TB-CM on skin fibroblasts. RNA-seq analysis identified 160 genes as significant differentially expressed genes (DEGs; <2-fold change) in the TB-CM-treated HNDFs. Ninety genes were significantly upregulated, while 70 genes were significantly downregulated (Figure 5A). The significantly upregulated and downregulated genes, with fold changes, have been represented in the form of a Volcano plot (Figure 5B). Panel C of Figure 5 shows the Top 30 upregulated and downregulated genes in the TB-CM-treated HNDF cells. Following this, the DEGs up- and downregulated by a fold change of 2-fold or greater in the TB-CM-treated HNDF cells were further subjected to gene ontology (GO) analysis. The key GO terms for biological processes included response to external biotic stimulus, cytokine-mediated signaling pathway, and defense response to viruses (Figure 6A). The GO terms for molecular function included C-X-C motif ligand (CXCL) chemokine receptor binding, receptor regulator activity, and signaling receptor activator activity (Figure 6A). Kyoto Encyclopedia of Genes and Genomes (KEGG) pathway analysis was carried out based on the DEGs in the TB-CM-treated HNDF cells, to define a more exact mechanism and pathway at the cellular level. Cytokine- and chemokine-related signaling pathways were also determined in the GO analysis result with virus defense mechanism (coronavirus disease (COVID-19)). Toll-like receptor (TLR) and nuclear factor kappa B (NF-κB) signaling pathways were associated with TB-CM treatment of scratch-wounded HNDFs. From the results of the GO and KEGG analyses, we selected the upregulated CXCL family genes in the TB-CM-treated HNDFs, confirmed their expression levels in vitro using quantitative real-time RT-PCR, and then compared the results obtained with the results of RNA-seq. Strong upregulation of *CXCL* family genes such as *CXCL1*, *CXCL6*, *CXCL8*, *CXCL10*, and *CXCL11* was observed in TB-CM-treated HNDF cells (Figure 6C), suggesting that TB-CM might induce chemokine-related cellular pathways during wound healing in skin fibroblasts.

## 4. Discussion

Among placental components, the regenerative properties of the amniotic membrane have been well-studied since 1910 and steadily developed for application in various reconstructive surgical procedures [25,26,27]. The bioactive factor-containing epithelial layer of the amniotic membrane must be decellularized to avoid unwanted immunological rejection by a recipient; therefore, this process reduces the regenerative efficacy of the amniotic membrane. In contrast to the amniotic membrane, the chorionic membrane compactly consists of three cellular layers, namely reticular, basement, and trophoblast layers, which warrants its use as a biological dressing for wound surface reconstruction [28]. The chorionic membrane plays an important role in forming the connection between the fetus and mother to facilitate mutual trafficking, not only for nutrition supplementation and gas exchange, but also for the removal of metabolic waste from the fetus. In addition, the chorionic membrane contains a better variety of bioactive factors such as collagen, cell-adhesion bioactive factors (fibronectin, lamin, and proteoglycan), and growth factors, compared to the amniotic membrane, which have beneficial effects on the overall functional properties of the placenta [28,29]. Therefore, the chorionic membrane or its composed cells can serve as excellent biomaterials for tissue engineering. Nonetheless, little is known about the functional applications of the chorionic membrane.

In the present study, we focused on the regenerative capacity of trophoblast cells in the chorionic membrane and revealed the protection/recovery effects of TB-CM and TB-Exos on skin aging due to intrinsically or extrinsically induced skin damage. The dermis layer in the skin includes fibroblasts, which synthesize fundamental components such as collagen, elastin, and fibronectin to constitute the extracellular matrix (ECM) [30]. Aged fibroblasts have conspicuously lower regenerative capacities for the synthesis of ECM, proliferation, and migration than young cells [31]. Increases in the levels of MMP, interleukin-6 (IL-6), angiogenic inducer protein, and in pro-apoptotic gene expression are also commonly observed in aged fibroblasts [32,33,34]. However, TB-CM and -Exos stimulated a decrease in MMP gene expression and an increase in collagen, elastin, and fibronectin gene expression in HNDFs. In particular, we observed that TB-CM and TB-Exo mediated proliferation and migration in HNDFs, indicating that some paracrine factors secreted from the trophoblasts in TB-CM and TB-Exos are clearly involved in the regeneration of skin fibroblasts. Overall, TB-derived CM and Exos exerted a rejuvenating effect and were not cytotoxic to skin fibroblasts, and in addition, induced a remarkable recovery of SA-β-Gal-stained cells to unstained cells, which are youthful cells in senescent HNDFs.

Cells usually secrete biological factors, such as cytokines, growth factors, other proteins, lipids, and nucleic acids to communicate with adjacent cells [35,36]. For example, stem cells produce and secrete various chemokines, which can modulate the migration of macrophages [37]. Chemokines play important roles in promoting wound healing, which dynamically regulate angiogenesis, inflammation, proliferation, and the remodeling stage of wound healing [38]. During migration of scratch-wounded HNDFs in our study, TB-CM treatment specifically led to the upregulation of *CXCL* chemokine genes, including *CXCL1, CXCL6*, *CXCL8*, *CXCL10*, and *CXCL11*, as identified using RNA-seq. It is known that chemokines regulate wound healing via growth factors [39,40,41]. Together with CXCL8- and transforming growth factor-beta-induced differentiation of fibroblasts into myofibroblasts, cells secrete various ECM components such as fibronectin, which suppress the integrin-mediated cell adhesion that facilitates cell movement for wound closure [40]. CXCL8 and CXCL1 have been shown to stimulate neo-angiogenesis and cell migration, while CXCL9, 10, and 11 have been shown to induce anti-inflammatory and angiogenesis at the proliferation stage of the wound healing process in previous studies [40,42]. A recent study reported that wound exudate CXCL6 levels were increased in the rapidly healing patients with neuropathic diabetic foot ulcers as a biomarker for wound healing [43]. In the remodeling stage of wound healing, excess neo-vessels are removed by CXCL10 and CXCL11 via preventing endothelial cells tubulogenesis [44]. Interestingly, we found increased expression levels of TLR3 and growth differentiation factor 11 (GDF11) in our study. Nelson et al. showed that double-stranded RNA from damaged tissue induced the activation of TLR3 to drive skin regeneration via IL-6 and signal transducer and activator of transcription 3-mediated wnt/shh signaling in hair follicles [45]. A recent study considered GDF11 as an anti-aging effector that can preserve the youthful phenotypes of several types of skin cells [46]. In addition, GDF11 is also associated with the inactivation of inflammatory responses and protection against them by inhibiting oxidative stress and the expression of heat shock proteins [47]. Downregulation of homeodomain interaction protein kinase 4 was determined in our analysis; its silencing was found to be related to skin epithelial differentiation from stem cells in a previous study [48].

Activation of the TLR and NF-κB signaling pathways promotes epidermal wound repair in *Drosophila* [49]. NF-κB signaling is capable of regulating proteins such as cyclin D1, fibronectin, and vascular endothelial growth factor, which leads to wound healing in rats [50]. We also found that TLR and NF-κB signaling were the most related cellular pathways upon treatment of scratch-wounded HNDFs with TB-CM, as determined using RNA-seq. The exact mechanism of TB-CM in HNDFs remains unknown, but our results suggest that TB-CM effectively promotes HNDF cell proliferation and migration by inducing chemokine expression and other regenerative activators.

We used a trophoblast cell line isolated from an embryo of 6 to 12 weeks gestation, and which could develop into a large proportion of the placenta, directly connect with maternal blood, and supply nutrients and oxygen to the fetus. This cell line expresses stem cell marker genes encoding for OCT4, Sox2, Nanog, and Klf4, whose expression levels were compatible with those observed in the MSCs in our study (data not shown). Cellular approaches using paracrine factors from stem cells, including embryonic stem cells, induced pluripotent stem cells (iPSCs), and MSCs have been used in regenerative medicine [16,51,52]. However, trophoblast stem cells (TSCs) have not yet been studied. TSCs can be obtained from discarded placenta using a recently established protocol [53] or from somatic cells and iPSCs [54,55]. If somatic cells or iPSCs are used to obtain TSCs as cell sources, patient-specific TSCs can be generated and applied in clinical research for regenerative medicine and tissue engineering, including skin regeneration.

In conclusion, our study determined the skin regenerative capacity of TB-derived CM and Exos. In addition, treatment of aged HNDFs with TB-CM or TB-Exos promoted HNDF cell rejuvenation, modulation of ECM component expression, and chemokine activation. Therefore, our study suggests that CM and Exos from TB can serve as excellent therapeutic agents against skin aging.

## Figures and Tables

**Table 1:** Sequences of oligonucleotide primers for quantitative RT-PCR.

Primer	Direction	Sequence (5′ to 3′)
*collagen type 1 (COL1)*	F	CAC AGA GGT TTC AGT GGT TTG G
	R	GCA CCA GTA GCA CCA TCA TTT C
*collagen type 3 (COL3)*	F	CTG AAA TTC TGC CAT CCT GAA C
	R	GGA TTG CCG TAG CTA AAC TGA A
*matrix metallopeptidase 1 (MMP-1)*	F	TTG AGA AAG CCT TCC AAC TCT G
	R	CTG CAA CAC GAT GTA AGT TGT A
*matrix metallopeptidase 3 (MMP-3)*	F	TGC TGC TCA TGA AAT TGG CC
	R	TCA TCT TGA GAC AGG CGG AA
*Fibronectin 1*	F	AAG ATT GGA GAG AAG TGG GAC C
	R	GAG CAA ATG GCA CCG AGA TA
*Elastin*	F	GGG TTG TGT CAC CAG AAG CA
	R	CAA CCC CGT AAG TAG GAA TGC
*C-X-C motif chemokine ligand 1 (CXCL1)*	F	AAG TGT GAA CGT GAA GTC CC
	R	GTC ACT GTT CAG CAT CTT TTC G
*C-X-C motif chemokine ligand 6 (CXCL6)*	F	AGA GCT GCG TTG CAC TTG TT
	R	GCA GTT TAC CAA TCG TTT TGG GG
*C-X-C motif chemokine ligand 8 (CXCL8)*	F	ACT GAG AGT GAT TGA GAG TGG AC
	R	ACA ACC CTC TGC ACC CAG TT
*C-X-C motif chemokine ligand 10 (CXCL10)*	F	TTC TAC GCT GTA CCT GCA TCA
	R	TTC TTG ATG GCC TTC GAT TC
*C-X-C motif chemokine ligand 11 (CXCL11)*	F	AGA GGA CGC TGT CTT TGC AT
	R	TAA GCC TTG CTT GCT TCG AT
*GAPDH*	F	TCG CCC CAC TTG ATT TTG G
	R	GCA AAT TCC ATG GCA CCG T

## Data Availability

Data is contained within the article or supplementary material.

## Supplementary Materials

The following are available online at https://www.mdpi.com/article/10.3390/ijms22136959/s1.

## Abbreviations

TB	trophoblasts
EV	extracellular vesicles
HNDFs	human normal dermal fibroblasts
TB-CM	trophoblast-derived conditioned medium
TB-Exo	trophoblast-derived exosomes
ECM	extracellular matrix
MSCs	mesenchymal stem cells
iPSCs	induced pluripotent stem cells
MSC-CM	MSC-derived conditioned medium
qRT-PCR	quantitative real-time polymerase chain reaction
CCK8	cell counting kit 8
MMP	matrix metalloproteinase
MSC-Exo	MSC-derived exsomes
SA-β-Gal	senescence-associated β-galactosidase
DEGs	differentially expressed genes
KEGG	Kyoto encyclopedia of genes and genomes
GO	gene ontology
TGF-β	transforming growth factor-beta
TLR3	toll-like receptor 3
GDF11	growth differentiation factor 11
IL-6	interleukin-6
STAT3	signal transducer and activator of transcription 3
NF-κB	nuclear factor kappa B
VEGF	vascular endothelial growth factor
HIPK4	homeodomain interaction protein kinase 4
ESCs	embryonic stem cells
TSCs	trophoblast stem cells
UVB	ultraviolet B
FBS	fetal bovine serum
PBS	phosphate-buffered saline
