# Supplementary material for: Extracellular Vesicles (Secretomes) from Human Trophoblasts Promote the Regeneration of Skin Fibroblasts
Source: Int J Mol Sci. 2021 Jun 28;22(13):6959. doi: 10.3390/ijms22136959 (PMC8269172; doi:10.3390/ijms22136959)
Supplement: Supplementary file 1 [file ijms-22-06959-s001.zip › ijms-1256395-supplementary.pdf]

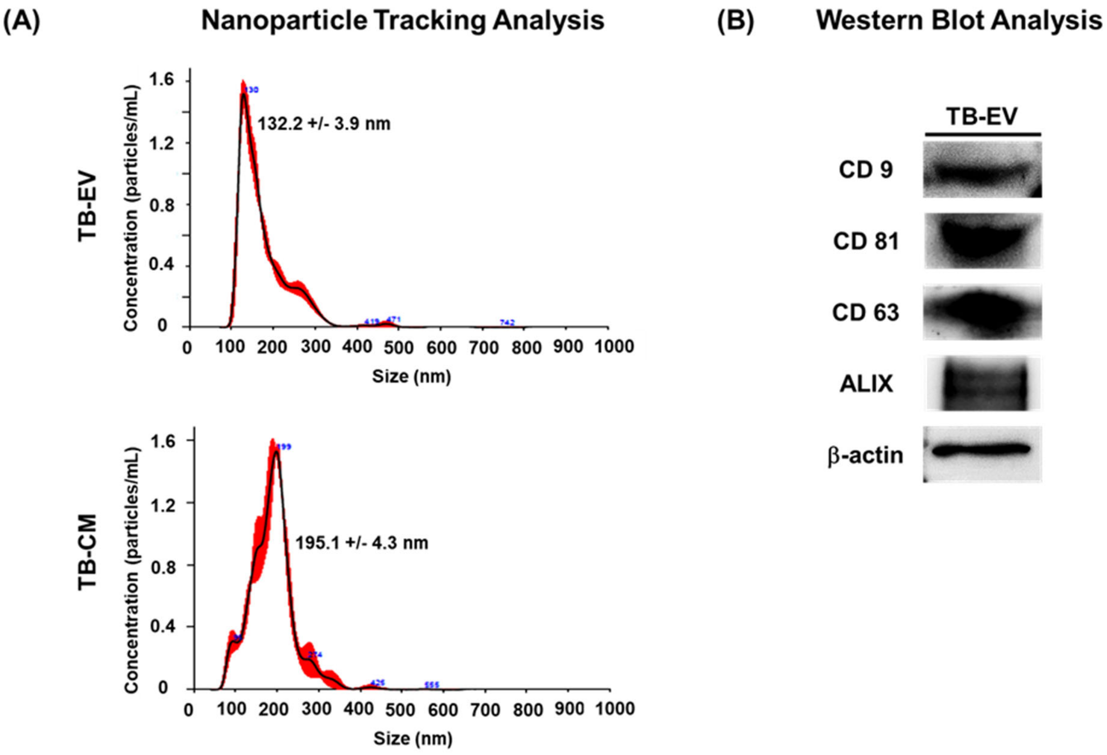

Supplementary Figure S1. Characterization of TB-CM and TB-Exos.**(A)** Representative nanoparticle tracking analysis results of TB-CM and TB-Exo are shown here. Different distributions and sizes of nanoparticles were observed in TB-CM and TB-Exos. The number on each graph indicates the average particle size. **(B)** Expression levels of exosome-specific markers such as CD9, CD81, CD63, and ALIX were determined in TB-Exos using western blot analysis.

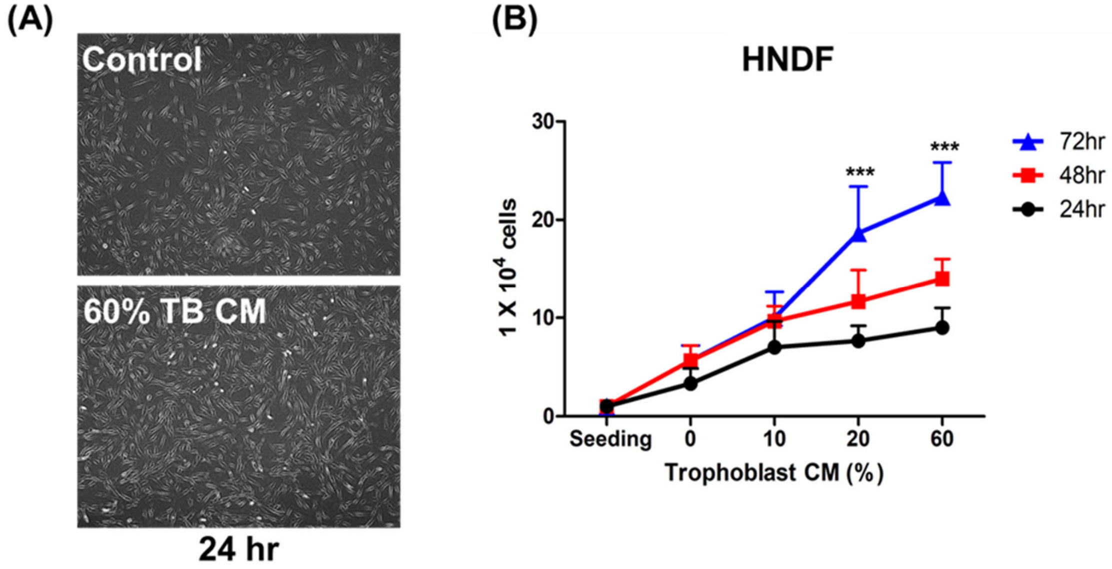

Supplementary Figure S2. Proliferation effect of TB-CM.**(A)** Representative images of HNDFs treated with 60%TB-CM for 24 h. **(B)** HNDF cells were treated with 0%, 10%, 20%, and 60% TB-CM for 24, 48, and 72 h, following which the live cells were counted using Trypan Blue stain with a hemacytometer.
